# Diagnostic Technologies for Urinary Retention in the Postoperative Period of Arthroplasties: An Integrative Review

**DOI:** 10.1155/nrp/1555094

**Published:** 2026-06-22

**Authors:** Carlos Alexandre dos Santos Farias, Priscilla Alfradique de Souza, Rosane Barreto Cardoso, Graziele Ribeiro Bitencourt, Carlos Henrique Terra Pereira

**Affiliations:** ^1^ Universidade Federal do Estado do Rio de Janeiro, Rio de Janeiro, Brazil, unirio.br; ^2^ Universidade Federal do Rio de Janeiro, Rio de Janeiro, Brazil, ufrj.br

**Keywords:** arthroplasty, diagnosis, review, technology, ultrasonography, urinary retention

## Abstract

**Aims:**

To identify diagnostic technologies for postoperative urinary retention in patients undergoing lower limb arthroplasty surgeries.

**Design:**

Integrative review.

**Data Sources:**

Searches were conducted in April 2024 in the following databases: Medical Literature Analysis and Retrieval System Online (MEDLINE), PubMed, Web of Science, Scopus, and CINAHL.

**Review Methods:**

Studies published in the last 10 years, without language restriction, were included. The search strategy combined the terms “postoperative,” “arthroplasty,” “diagnosis,” and “urinary retention.” Study selection and analysis followed PRISMA recommendations. Data were analyzed descriptively and synthesized qualitatively.

**Results:**

A total of 34 studies were included, comprising 36,736 participants. Bladder ultrasound was recommended in 73.5% of the studies as the primary diagnostic technology. The most frequent urinary volume thresholds indicating catheterization ranged from 400 to 500 mL, with postvoid residual volumes around 150 mL also reported.

**Conclusion:**

Bladder ultrasound is the most recommended diagnostic technology for postoperative urinary retention in patients undergoing lower limb arthroplasty. However, further studies are needed to validate its use across different clinical contexts and populations.

**Impact:**

The findings support the incorporation of bladder ultrasound into clinical protocols to improve early diagnosis, reduce unnecessary catheterization, and enhance patient safety in postoperative care. This review also highlights the need for greater involvement of nursing professionals in research and clinical application of diagnostic technologies.

**Patient or Public Contribution:**

Not applicable.

## 1. Introduction

Hip and knee arthroplasties are among the most frequently performed orthopedic procedures and represent a significant proportion of the global surgical landscape. According to the Organisation for Economic Co‐operation and Development (OECD), in its 2021 report, countries such as Switzerland, Germany, Austria, and Finland reported the highest rates of hip arthroplasty, with 321, 301, 287, and 284 procedures per 100,000 inhabitants, respectively. For knee arthroplasties, Switzerland, Finland, Australia, and Germany presented the highest rates, with 273, 260, 252, and 201 procedures per 100,000 inhabitants, respectively [1]. In Brazil, data from the Ministry of Health indicate that 16,235 primary total knee arthroplasties and 28,739 primary total hip arthroplasties were performed between 2018 and 2019 [2].

Patients undergoing these elective lower limb orthopedic procedures may experience several postoperative complications, one of the most common being postoperative urinary retention (POUR) [3–5]. POUR is defined as the inability to voluntarily empty the bladder after anesthesia and surgery, resulting in bladder overdistension [6, 7]. This condition is frequently reported as an adverse event following hip and knee arthroplasty procedures [6].

The incidence of POUR in postoperative care units is estimated at approximately 16%, but it may reach up to 75% in patients undergoing lower limb arthroplasties [5–7]. In a univariate analysis with 313 patients, risk factors for this phenomenon were observed to be age over 50 years, major surgeries, surgery duration greater than 60 min and anesthesia duration greater than 80 min, administration of fluids greater than 750 mL intraoperatively, and bladder volume upon admission to the postoperative unit greater than 270 mL [5]. Another study evaluated the prevalence and predictive factors of POUR, specifically including lower limb surgeries as one of these factors [8].

Early diagnosis of POUR is essential to prevent complications and optimize postoperative recovery. Bladder overdistension may lead to detrusor muscle ischemia, oxidative damage, and apoptosis, potentially resulting in permanent bladder dysfunction even after a single episode [9]. In addition, delayed diagnosis may lead to acute kidney injury and prolonged hospitalization [10].

The occurrence of acute urinary retention events in the immediate postoperative period of arthroplasties is frequent. A systematic review mapped 15 articles published between 2010 and 2019 and found that in patients undergoing hip and knee arthroplasties the incidence of POUR can reach 46% [11].

Nursing assessment plays a key role in the early identification of POUR, aiming to prevent both delayed and unnecessary catheterization. However, there is no consensus regarding the optimal bladder volume threshold for intervention, with values ranging from 400 to 600 mL reported in the literature [12–16].

Inadequate management of urinary retention after surgery can lead to delays in surgical recovery, the start of the rehabilitation process, and reintegration into the workforce, as well as increased hospital stays. Evidence shows that patients who require an indwelling urinary catheter after an episode of POUR have a significant decrease in walking distance after total knee arthroplasty compared to patients who were able to urinate spontaneously during this postoperative period. Subsequently, this also increases the risk of deep vein thrombosis [17].

Urinary control is inhibited after the anesthetic event. Therefore, to assess an individual’s ability to regain sphincter control and consequently, regain the perception of bladder fullness and complete emptying, knowledge of the pharmacodynamics of the chosen sedative is also necessary. Opioids, for example, increase the tone and amplitude of urinary sphincter contractions and decrease ureteral contractions, thus hindering spontaneous urination [18]. Preoperative conditions such as urinary symptoms, need for bladder catheterization in previous surgeries, obesity, surgical type (bilateral hip, for example) are important information to be verified when collecting the patient’s history by the nurse, as they may indicate an increased risk of spontaneous voiding difficulties in the postoperative period. However, clinical observation may not be sufficient for diagnostic confirmation, requiring the support of technological tools.

In this sense, diagnostic technologies are essential tools to assist nurses in the care of patients with urinary retention. They are also particularly useful in preventing unnecessary bladder catheterizations, improving the quality of care, and personalizing the assessment of patients with retention. In this way, they can enhance diagnostic accuracy by corroborating the indication of the best time for bladder catheterization when the patient is unable to voluntarily empty their bladder [19].

## 2. Objective

To identify diagnostic technologies for postoperative urinary retention in lower limb arthroplasty surgeries (hip and knee).

## 3. Methods

### 3.1. Ethical Aspects

Because this is an integrative review, the research is exempt from the need for submission to and approval by the Research Ethics Committee.

### 3.2. Study Design

This is an integrative literature review conducted through six stages: identification of the research problem or question; literature search based on defined criteria; data collection; critical appraisal of the included studies and classification of the level of evidence; interpretation and synthesis of results; and presentation of knowledge [20, 21]. The review followed the PRISMA guidelines (Preferred Reporting Items for Systematic Reviews and Meta‐Analyses) to ensure transparency in study selection [22].

### 3.3. Identifying the Research Question

The integrative review question was developed using the PICo [22] strategy (Population, Interest, Context). This considered the following: Population (P)–patients undergoing knee or hip arthroplasty; Interest (I)–diagnostic technologies for urinary retention; Context (Co)–postoperative period. This led to the following guiding question: “What diagnostic technologies are used to identify urinary retention in patients undergoing knee or hip arthroplasty in the postoperative period?”

### 3.4. Inclusion and Exclusion Criteria

The inclusion criteria were defined as original research and review articles available in full, without language restrictions, from the last 10 years due to the updating of terminology, and the exclusion criteria were letters, editorials, books, abstracts from conference proceedings, theses, and dissertations.

### 3.5. Research Strategy

The search was conducted in the following databases: Medical Literature Analysis and Retrieval System Online (MEDLINE), PubMed, Web of Science, Scopus, and CINAHL. The search strategy combined controlled descriptors and keywords related to the topic, including “postoperative,” “arthroplasty,” “urinary retention,” and “diagnosis.” Boolean operators (AND, OR) were used to refine the search and ensure comprehensive retrieval of relevant studies.

An example of the search strategy used in PubMed was (“postoperative” AND “arthroplasty” AND “urinary retention” AND (diagnosis OR ultrasonography OR “bladder scan”)).

The search was conducted in April 2024. After full‐text reading, the level of evidence of the included studies was assessed and classified according to the Oxford Centre for Evidence‐Based Medicine (OCEBM) levels of evidence.

### 3.6. Data Extraction and Analysis

The selected studies were organized for initial screening of titles and abstracts, followed by the removal of duplicate records. Titles and abstracts were independently assessed by two reviewers according to the inclusion criteria. Potentially relevant studies were retrieved in full text. The full‐text articles were then analyzed in detail by two independent reviewers to confirm eligibility. Studies that did not meet the inclusion criteria were excluded. In cases of disagreement at any stage of the selection process, consensus was reached through discussion or consultation with a third reviewer.

A data extraction tool was used to systematically collect information from the included studies. Diagnostic technologies were defined as tools or methods used to support the identification or confirmation of urinary retention, including imaging devices, clinical assessment criteria, and monitoring strategies. The extracted data included title, authors, year of publication, country, study setting, authors’ professional background, central theme, methodological design, level of evidence, and variables related to the diagnosis of urinary retention in the postoperative period of hip and knee arthroplasties, such as incidence, complications, and reported urinary volumes.

Descriptive statistical analysis was performed to characterize the studies included in the final sample. The studies were analyzed descriptively and synthesized qualitatively, allowing for critical interpretation of the findings. The main evidence regarding the diagnosis of urinary retention in the postoperative period of lower limb arthroplasties was identified and compared across studies. Patterns, inconsistencies, and gaps in the use of diagnostic technologies were also explored. The data are presented in tabular form, and qualitative summaries were used to complement and interpret the findings.

## 4. Results

**FIGURE 1:**
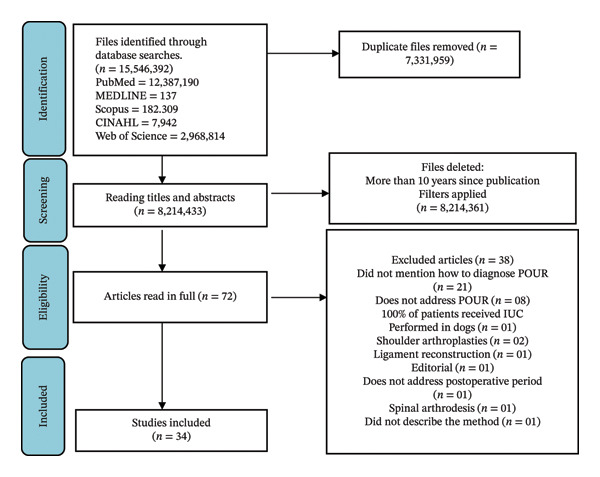
Flowchart showing the distribution of the number of articles found, excluded, and selected by database according to PRISMA. Rio de Janeiro, RJ, Brazil, 2024.

A total of 149 records were identified across the databases. After the removal of duplicates and initial screening, 129 records underwent title and abstract review, of which several were excluded. Subsequently, 72 full‐text articles were assessed for eligibility, and 34 studies were included in the final sample, as shown in Figure 1.

Table 1 presents a summary of the consolidated data from the studies selected in this review, categorized by year of publication.

**TABLE 1:** Characteristics of included studies: year, authors, country, study design, level of evidence, sample size, diagnostic criteria for postoperative urinary retention (POUR), incidence, mean age, and diagnostic bladder volume.

Authors/country/year	Study design and level of evidence sample number (*n*)	Diagnostic criteria for POUR Bladder volume (V) for diagnosis	Incidence of POUR (%) Mean age (years)
Hejkal et al./United States/2023 [23]	Controlled cohort prospective—2b *n*: 1397	Use of the US V: > 300 mL with discomfort or > 500 mL	13% not reported
Dana et al./Israel/2023 [24]	Retrospective cohort—2b *n*: 105	Bladder catheterization V: It was not mentioned.	21% 18 patients > 65 years old and 4 patients under 65 years old
Weintraub et al./United States/2023 [25]	Randomized clinical trial—1b *n*: 388	Use of the US V: > 450 mL	2.1% in the Foley group and 2.6% in the control group. Not reported
Kotodziej et al./Poland/2023 [26]	Prospective observational study—2c *n*: 53	Use of ultrasound or bladder distension causing discomfort and pain. V: > 500 mL	51% not reported
Magnuson et al./United States/2023 [27]	Randomized clinical trial—1b *n*: 728	Use of the US V: > 500 mL in the control group > 600 mL in the elective group	15% control group, 14.8% selective group not reported
Hoffmann et al./Netherlands/2022 [28]	Longitudinal cohort prospective—2b *n*: 489	Bladder catheterization V: It was not mentioned.	Tourniquet group 3% and control group 0.9% Not reported
Peng et al./China/2022 [29]	Controlled cohort prospective—2b *n*: 381	Use of the US V: > 500 mL	5.5% 61.3
Ding et al./China/2022 [30]	Randomized controlled clinical trial—1b *n*: 170	Use of the US V: > 400 mL	17.6% doxazosin group, 36.5% control group not reported
Choi et al./South Korea/2021 [31]	Prospective randomized controlled trial—1b *n*: 95	Use of the US V: > 400 mL	12.5% in the tamsulosin group and 29.8% in the control group. Not reported
Thiengwittayaporn et al./Thailand/2021 [32]	Prospective randomized controlled trial—1b *n*: 230	6 h without urinating and suprapubic discomfort V: > 400 mL	2.6% and 5.3% in the control group. Not reported
Bracey et al./United States/2021 [33]	Prospective cohort—2b *n*: 271	Use of the US V: > 400 mL	20.00% 63
Abdul‐Muhsin et al./United States/2020 [34]	Prospective controlled cohort—2b *n*: 1374	Use of the US V: It was not mentioned.	9.02% 72.35
Cha et al./South Korea/2020 [11]	Systematic review—1st *n*: 6397	Use of ultrasound in the 15 studies V: 300 to 700 mL	4.1% to 46.3% not reported
Garbarino et al./United States/2020 [35]	Prospective cohort—2b *n*: 9123	Intermittent or prolonged bladder catheterization V: It was not mentioned.	13.00% 67.9 IUC; 67.5 intermediate; 70.9 intermediate and IUC
Gold et al./United States/2020 [17]	Prospective cohort—2b *n*: 9123	Intermittent or prolonged bladder catheterization V: It was not mentioned.	13.07% 67.9 IUC; 67.5 interm; 70.9 interm and IUC
Santini et al./United Kingdom/2019 [36]	Prospective cross‐sectional study—2c *n*: 303	Bladder catheterization V: It was not mentioned.	8.60% not reported
Schubert et al./United States/2019 [37]	Randomized clinical trial—1b *n*: 131	Use of the US V: It was not mentioned.	32.10% not reported
Kwak et al./South Korea/2019 [38]	Retrospective cross‐sectional study—2c *n*: 214	Use of ultrasound or bladder catheterization V: It was not mentioned.	31.80% 78.70
Ziemba‐Davis et al./United States/2019 [39]	Retrospective cross‐sectional study—2c *n*: 633	Intermittent bladder catheterization V: It was not mentioned.	5.50% not reported
Markopoulos et al./Greece/2018 [40]	Randomized controlled clinical trial—1b *n*: 218	Use of the US V: It was not mentioned.	2.6% to 5.8% not reported
Scholten et al./Netherlands/2018 [6]	Prospective observational study—2c *n*: 381	Use of the US V: It was not mentioned.	46.30% not reported
Halawi et al./United States/2018 [41]	Retrospective cross‐sectional study—2c *n*: 378	Use of the US V: It was not mentioned.	38.00% 63.10
Mahan et al./United States/2018 [42]	Prospective cohort study—2b *n*: 156	Use of the US V: It was not mentioned.	3.8% with mepivacaine and 16.5% with bupivacaine. Not reported.
Kort et al./Netherlands/2017 [7]	Retrospective cohort—2b *n*: 638	Use of the US V: It was not mentioned.	12.90% 68.64
Lawrie et al./United States/2017 [43]	Prospective cohort—2b *n*: 174	Use of the US V: It was not mentioned.	43.70% 67
Tischler et al./United States/2016 [44]	Prospective cohort—2b *n*: 842	Use of the US V: It was not mentioned.	9.30% not reported
Rana et al./United States/2016 [45]	Prospective cohort—2b *n*: 51	Use of the US V: It was not mentioned.	19.60% 69
Bjerregaard et al./Denmark/2015 [46]	Prospective observational study—2c *n*: 1062	Use of ultrasound or bladder catheterization if symptomatic urinary retention is present. V: 400 to 600 mL, median of 550 mL	40.40% 68
Huang et al./China/2015 [47]	Randomized controlled trial—1b *n*: 314	Use of the US V: > 400 mL	6% in total, with 5.7% in the control group and 6.4% in the group that used a probe. 75.20
Kasture et al./India/2015 [48]	Randomized controlled—1b *n*: 75	5 cm increase in abdominal circumference with discomfort. V: It was not mentioned.	20% epidural anesthesia and 5% intra‐articular anesthesia. Not reported
Fernandez et al./United Kingdom/2014 [49]	Retrospective cohort—2b *n*: 420	Absence spontaneous urination within 7 h after anesthesia and with symptoms; a urine volume > 400 mL at the time of urethral catheterization. V: > 400 mL	General anesthesia + BNP: 14% h 2%; spinal anesthesia + intrathecal morphine: 9% h 3% m; spinal anesthesia + intrathecal morphine: 60% h 5% m Not reported
Nyman et al./Sweden/2013 [50]	Randomized clinical trial—1b *n*: 170	Use of the US V: 400 mL or postvoid residual urine greater than 150 mL	Not applicable or 86% in the intermittent group71.9 intermittent group and 72.1 in the IUC group
Miller et al./United States/2013 [51]	Prospective randomized controlled trial—1b *n*: 93 (group without catheter) and 107 (group with catheter)	Use of the US V: > 400 mL	9.7% (group without catheter) and 2.8% (group with catheter) 55.50
Karason et al./Iceland/2013 [52]	Prospective observational study—2c *n*: 52	Use of the US V: > 400 mL	12.00% 69.00

*Note:* Rio de Janeiro, Brazil, 2024 (*n* = 34). BNP—peripheral nerve block; FTN: fentanyl; POUR—postoperative urinary retention; US—ultrasound.

Abbreviations: IUC, indwelling urinary catheterization; UTI, urinary tract infection.

The analysis of the results of this review revealed a robust profile of research related to diagnostic technologies for urinary retention, with 68% (*n* = 24) of the articles published in the last 5 years, showing an increase in the number of publications on the subject compared to the previous 5 years. Regarding the publication locations, the United States stands out with 15 publications–representing 44% of the works, 11 publications in Europe—32%, and 8 publications originating from Asia–totaling 24%. Regarding the authors, 91% of the publications were produced by medical professionals, with 73% being orthopedists; and of the total, only 6% were authored by nurses. The Journal of Arthroplasty was the journal with the highest number of occurrences in this review—13 articles (38%), followed by Journal of Orthopedic Surgery with 3 articles (9%). Only 1 journal specifically in the field of Nursing appeared in this selection—the Journal International of Nursing Studies—with 2 articles.

Regarding the methodology employed in the selected articles, cohort studies predominated, accounting for 41% (*n* = 14) of the studies, followed by 32% (*n* = 11) of randomized controlled clinical trials, 24% (*n* = 08) of cross‐sectional observational studies, and 3% (*n* = 01) of systematic reviews.

Regarding the quality of the journals from which the articles for this review were selected, it was observed that using the Scimago—SCOPUS H Impact Factor analysis, 59% (*n* = 20) of the studies had a score greater than 100. Of the journals studied, 65% (*n* = 22) are in Q1—which represents 75%–100% of the best journals on the topic; Using the Journal Citation Reports (JCR)—2022 edition, the impact factor ranged from 1.4 to 8.1.

Additional characteristics related to authorship, publication region, journal profile, and level of evidence are presented in Table 2.

**TABLE 2:** Description of the profile of the articles included in the study.

Characteristics of the studies		N	%
Year of publication	2013–2017	11	32
	2018–2023	23	68

Publication region	Europe	11	32
	Asia	08	24
	United States	15	44

Professionals	Orthopedic doctor	25	73
	Urologist	02	06
	Anesthesiologist	03	09
	Geriatrician	01	03
	Nurse	02	06
	Director of research	01	03

Central theme of the magazine	Orthopedics	10	29
	Arthroplasty	13	38
	Anesthesia	03	09
	Nursing	02	06
	Others	06	18

Study type: classification of the level of evidence by study type “*Oxford Center for Evidence—Based Medicine* ”	1A	01	03
	1B	12	35
	2B	13	38
	2C	08	24

Impact factor H SCIMAGO 2020	0–100	13	38
	101–200	18	53
	201	02	06
	Journal not yet rated	01	03

SCIMAGO factor quartile	Q1	22	65
	Q2	05	15
	Q3	06	18
	Journal not yet rated	01	03

JCR 2022 impact factor	0–3	14	41
	3.01–8.1	19	56
	Journal not yet rated	01	03

*Note:* Rio de Janeiro, RJ, Brazil, 2024. (*n* = 34).

Abbreviation: JCR, Journal Citation Reports.

The included studies reported different diagnostic approaches for the identification of postoperative urinary retention, including point‐of‐care bladder ultrasound, bladder catheterization, clinical assessment parameters (such as time without voiding and suprapubic discomfort), and indirect indicators such as abdominal distension. Point‐of‐care bladder ultrasound was identified as the main diagnostic technology for the diagnosis of urinary retention in the postoperative period. Other technologies described for the diagnosis of urinary retention were bladder catheterization in 17% (*n* = 9) of the studies; a 5‐cm increase in abdominal circumference associated with complaints of discomfort [48]; 6 h without urinating and with complaints of suprapubic discomfort [32]; absence of spontaneous urination within 7 h after anesthesia and with symptoms of urinary retention [25]; and bladder distension causing discomfort and pain [49]^.^

The incidence of POUR presented in the studies varied between 0.9%, found in the control group of one study [38], to 60% in men undergoing spinal anesthesia and intrathecal morphine described in another study [46]. Considering all the studies, there is an average incidence of 23.21%.

Another diagnostic tool highly regarded in the studies was urine volume. This was used to indicate the need for bladder drainage, being presented in 76% (*n* = 26) of the studies, with the following main recommendation values: the vast majority, 73% (*n* = 19), recommend bladder catheterization with a volume between 400 and 500 mL and an average of 437.7 mL. In addition, the other studies mentioned a high variability in the volumes considered. One study considered a volume greater than 600 mL [7, 27]; a systematic review [11] presented the range between 300 and 700 mL; another study [30] also presented the range between 400 and 600 mL (median of 550 mL); one study [28] presented an average of 666 mL as a parameter to indicate catheterization; another study [42] used a volume of 240 mL; One study [41] used a volume of 350 mL. And lastly, a volume of 300 mL [23] was indicated if it is associated with discomfort and if it is spontaneous with an 8‐h interval in another study [39].

In 4 studies (14%), another criterion was also included to support the diagnosis of urinary retention. Postvoid urinary volume was mentioned and measured, considering the need for catheterization when it presents a volume greater than 200 mL [37, 39] and 150 mL [6, 50].

## 5. Discussion

The use of technologies to support the diagnosis of postoperative urinary retention is an important strategy in the management of patients undergoing orthopedic surgeries, and arthroplasties are highlighted in this study. Physical examination of the urinary bladder can be considered a low‐level technology to aid in this diagnosis. However, it does not offer the evaluator the necessary sensitivity to obtain a precise measurement of the urinary volume retained in the organ [16].

The results found in this integrative review show that the vast majority of studies found confirm the accurate diagnosis of urinary retention using point‐of‐care ultrasound (POCUS). These findings corroborate the publication by the National Health Surveillance Agency (ANVISA, 2017), which highlights ultrasound as a strategy to prevent catheter‐associated urinary tract infections (CAUTI), avoiding unnecessary catheterization and supporting protocols for postoperative urinary retention management [53]. No clinical practice guidelines or consensus statements specifically addressing diagnostic technologies for postoperative urinary retention in arthroplasty were identified in this review, highlighting a gap between available evidence and standardized clinical recommendations.

Just as important as the nurse’s identification of urinary retention is the safe identification of the volume present inside the bladder. This allows for determining whether immediate emptying is necessary. Therefore, a management protocol is important to guide actions, ensuring the best interval between assessments and preventing bladder distension or premature catheterizations. Delayed diagnosis of urinary retention can lead to bladder distension with acute kidney injury and detrusor muscle injury [12], which can increase length of stay and hospitalization, as well as hospital costs.

From this review, with a high number of findings, values between 400 and 500 mL also emerged as the most cited to indicate bladder catheterization, present in the vast majority of studies, demonstrating the robustness of the consensus. This information is extremely important for determining the cutoff point in the decision to catheterize by the nurse [54].

On the other hand, among the selected studies, there is a lack of national studies on the subject, as well as little direct participation of nurses in the publications, demonstrating the need for greater development in the area, since nurses frequently act in the diagnostic identification of urinary retention at the bedside.

It is worth noting that numerous publications from regional and federal nursing councils related to the use of ultrasound by nurses have legitimized its use in clinical practice, highlighting the objectives achieved with the use of this practice. Resolution 679/2021 [55] approves the standardization of performing bedside and prehospital ultrasound by nurses, as well as COREN‐SP opinion 029/2014 [56] which deals with the use of ultrasound by nurses for calculating volume in urinary retention, and COREN‐SC opinion 002/2020 [57] on the management of urinary retention with urinary residual assessment by ultrasound by nurses.

The use of ultrasound for other purposes is also highlighted, as in COFEN Resolution 627/2020 [58], which standardizes the performance of obstetric ultrasound by obstetric nurses and the opinions of COREN‐SP 003/2009 [59] on the performance of vascular ultrasound by nurses, and COREN‐RS 096/2013 [60] and COREN‐SC 28/2015 [61], which standardize the use of ultrasound for PICC insertion.

### 5.1. Study Limitations

As a limitation of this study, POUR was evaluated exclusively in the postoperative period, as this is the period in which this phenomenon occurs most frequently. It is recommended that further studies be conducted to verify its usability in clinical patients or those undergoing other surgical procedures.

### 5.2. Contributions to the Fields of Nursing, Health, or Public Policy

This study contributes to nursing, health, and public policy by highlighting bladder ultrasound as an effective tool in the early diagnosis of postoperative urinary retention, reducing complications, infections, and unnecessary catheterizations. The findings reinforce the importance of technology in healthcare and point to the need for updated protocols and professional training, promoting greater safety and optimization of clinical management.

## 6. Final Considerations

Bladder ultrasound has emerged as the primary diagnostic technology for postoperative urinary retention in patients undergoing lower limb arthroplasty due to its ability to provide accurate and non‐invasive assessments. This finding is supported by the evidence synthesized in this review, in which the majority of included studies recommended its use as the main diagnostic tool. When combined with clinical nursing practice, this technology enables more accurate diagnoses and safer interventions, contributing to the prevention of complications such as bladder distension, kidney injury, and prolonged hospital stay.

Although there is a general consensus regarding urinary volume thresholds for catheterization, typically between 400 and 500 mL, the results revealed variability across studies, indicating a lack of standardized diagnostic criteria. In addition, the limited participation of nurses in the included studies highlights a gap in nursing‐led research and in the evaluation and implementation of diagnostic technologies in postoperative care. These findings reinforce the need for greater involvement of nursing professionals in both research and clinical decision‐making processes, as well as the development of standardized protocols to support evidence‐based practice.

## Funding

No funding was received for this manuscript.

## Conflicts of Interest

The authors declare no conflicts of interest.

## Data Availability

Data sharing is not applicable to this article as no datasets were generated or analyzed during the current study.
